# County-level characteristics as predictors of dentists’ ECC counseling in the USA: a survey study

**DOI:** 10.1186/1472-6831-13-23

**Published:** 2013-05-20

**Authors:** Peter Milgrom, Colleen E Huebner, Lloyd A Mancl, Donald L Chi, Gayle Garson, David Grembowski

**Affiliations:** 1 Department of Oral Health Sciences, University of Washington, Box 357475, Seattle, WA, 98195-7475, USA; 2 Department of Health Services, University of Washington, Box 357230, Seattle, WA, 98195-7230, USA

**Keywords:** Early Childhood Caries, Prevention, Pregnant woman, County factors, Area Resource File

## Abstract

**Background:**

Transmission of *Streptococcus mutans* from mother-to-child can lead to Early Childhood Caries. A previous study identified characteristics and beliefs of general dentists about counseling pregnant women to reduce risk of infection and Early Childhood Caries. This study extends those findings with an analysis of county level factors.

**Methods:**

In 2006, we surveyed 732 general dentists in Oregon, USA about dental care for pregnant women. Survey items asked about individual and practice characteristics. In the present study we matched those data to county level factors and used multinomial logistic regression to test the effects of the factors (*i.e.*, dentist to population ratio, percentage of female dentists, percentage of females of childbearing age, and percentage of individuals living in poverty) on counseling behavior.

**Results:**

County level factors were unrelated to counseling behavior when the models controlled for dentists' individual attitudes, beliefs, and practice level characteristics. The adjusted odds ratios for no counseling of pregnant patients (versus 100 percent counseling) were 1.1 (95% CI .8-1.7), 1.0 (1.0-1.1), 1.2 (.9-1.5), and 1.1 (1.0-1.2) for dentist/population ratio, percent female dentists, percent females of childbearing age, and percent in poverty, respectively Similar results were obtained when dentists who counseled some patients were compared to those counseling 100 percent of patients.

**Conclusions:**

Community level factors do not appear to impact the individual counseling behavior of general dentists in Oregon, USA regarding the risk of maternal transmission of Early Childhood Caries.

## Background

*Streptococcus mutans* (SM), the primary bacteria responsible for the caries process [1], are not detectable in the human newborn [2]. Colonization happens early in life, even before the eruption of the first tooth [3]. The most common route and source of infection is through the exchange of salivary fluids with the infant’s primary caregiver, usually the mother [4]. Early colonization and high colonization levels of SM are associated with greater prevalence of early caries [5]. For these reasons, pregnant women and mothers of young infants should receive counseling regarding the process and prevention of early childhood caries. The Guideline on Perinatal Oral Health Care of the American Academy of Pediatric Dentistry provides an outline of recommended topics to discuss with patients [6].

In an earlier paper [7] we reported that the characteristic most strongly predictive of dentists’ counseling the majority of their pregnant patients about Early Childhood Caries (ECC) was dentists’ routine discussion of ECC with their office staff (OR=2.7, 95% CI 1.7-4.3). Additionally, the odds of providing counseling on ECC to the majority of pregnant patients was lower for male dentists than for female dentists (OR=0.53, 95% CI .29-.96). Among beliefs related to counseling behavior, the strongest predictor was dentists’ assessment of the strength of the published evidence for a link between caries in the mother and her infant (OR=1.6, 95% CI 1.2-1.9).

A number of studies from medicine have identified area-level variations in the provision of health care services [8-10]. A recent publication from dentistry examined county-level health profession workforce characteristics (i.e., percentage of female physicians, obstetricians or gynecologists, female dentists) as potential correlates of dentists’ counseling pregnant patients about prevention and periodontal disease risk [11]. That study did not find significant associations between county-level workforce characteristics and dentists’ counseling behavior. However, the study was based on a knowledge-building framework [12] that is relevant to the current investigation of the relation between county-level workforce characteristics and dentists’ counseling about ECC.

As applied to dentistry, the knowledge-building framework posits that dental practice innovation results from dynamic interactions between dentists and their social context, including other dentists with whom knowledge sharing and transfer can take place. Furthermore, the diffusion of innovation theory posits that patient-level needs, communicated through interpersonal networks comprised of individuals from similar backgrounds, also influence demand for health services [13]. This collective demand for care contributes to the social context of a community and is hypothesized to influence dentists’ knowledge building and counseling behaviors.

In this paper, we report the extent to which dentists’ counseling behavior about ECC is influenced by community level factors. Based on the knowledge- building framework and the diffusion of innovation theory, we hypothesized a higher proportion of patients would be counseled by dentists who practice in counties in which there were more dentists, a greater proportion of women dentists, more women of child-bearing age, and higher rates of poverty. The dentist-to-population ratio was included because it was hypothesized that a greater concentration of dentists would result in more sharing of new information among dentists. The percent of women dentists was included in our analyses because we presumed women would be more sensitive to the needs of other women and because of our previous finding that women dentists reported more frequent counseling than did male dentists [7]. The proportion of females of childbearing age and the proportion of persons living in poverty were included because both represent greater need and demand for dental care.

## Methods

### Survey population, materials, and methods

The study design is an analysis of a survey completed by general dentists in Oregon, USA [14]. The cross-sectional mixed-mode (mail or Internet) survey used the Tailored Design Method to maximize response rates and avoid non-response error in survey research [15]. Specific procedures included a stamped return envelope with the survey, a series of planned contacts with all intended recipients, the provision of a financial incentive in the same mailings as the survey itself, and personalized correspondence. Questionnaires were mailed in December 2006 to all 1,604 individuals identified through the master file of the American Dental Association as general dentists with practices in the State of Oregon. We restricted the mailing to practitioners in solo or partnership practices, associates in incorporated or unincorporated practices, or employees in state or government-run clinics. There were two mailed follow-ups.

The survey contained a total of 54 questions developed by the study team or drawn from previous studies [16-18]. Specific questions requested demographic information about the dentists and their patients and assessed dentists’ attitudes, beliefs, and practices regarding providing counseling to pregnant patients about caries transmission from mother to child. We also asked about practice ownership, professional training and experience, recent continuing dental education (CDE) and whether the dentist was an early or late adopter of new procedures in their practice of dentistry. The questionnaire is available for downloading from the website of the Northwest Center to Reduce Oral Health Disparities at http://depts.washington.edu/nacrohd/OR_Provider_Survey.

In the present study, we combined the survey data with four county level factors selected from the Area Resource File (ARF), a national dataset comprised of county-level health information [19]. We chose the following factors for 2007, the year of the survey of dentists: the ratio of dentists to the county population, percentage of women dentists, percentage of females of childbearing age, and percentage of individuals living in poverty 2007. The county variables associated with each dentist’s mailing address was used to link survey and ARF data.

The Institutional Review Board of the University of Washington approved the study. The elements of informed consent were contained in the cover letter.

### Analysis

Given that more than half the dentists either did no counseling or reported counseling 100 percent of their pregnant patients, we created three groups for purposes of analyses: dentists who provided no counseling about ECC (0%) to pregnant patients, dentists who counseled some (1-99%) pregnant patients, and those who counseled all pregnant patients (100%). Descriptive statistics were used to describe the sample, and chi-square tests and one-way ANOVA were used to determine how the provision of counseling was related to the county-level factors as well as to factors identified previously as correlates of dentists’ counseling women about periodontal health: dentists’ personal characteristics, practice characteristics, beliefs and attitudes, and whether the dentist was an early or late adopter of new procedures. We used multinomial logistic regression to identify the effects of county-level factors (dentist to population ratio, percentage of female dentists, percentage of females of childbearing age, and percentage of individuals living in poverty) independent of individual and practice level characteristics. Because our interest is in a population-averaged effect of individual and dental practice factors on dentist counseling on ECC among a simple random sample of Oregon general dentists, a multi-level analysis was not conducted. SAS (Version 9.2, SAS Institute Inc., Cary, NC) was used to conduct the statistical analyses.

## Results

### Survey respondents and characteristics of their dental practices

The completion rate among eligible survey participants was 55.2 percent (829/1502 individuals). The dentists practiced in 30 of 36 Oregon counties. There were no systematic differences between respondents and non-respondents in age or years since graduation from dental school. About 77 percent of the respondents graduated from the single dental school in Oregon, located in Portland. The current study includes the 732 of the 829 respondents who answered the question about ECC counseling and who provided sufficient geographic information about themselves to link their responses to their county-level characteristics. Table 1 gives the personal and practice characteristics of the respondents included in the present study.Early Childhood Caries.

**Table 1:** **Individual and practice characteristics of dentist respondents in Oregon State, USA (*N*= 732)**

**Sociodemographics**	**No.**^**1**^**(%) or Mean ± SD**
Age (Years)	46.4 ±11.9
Sex	
Male	601 (82.1)
Female	131 (17.9)
Years since receipt of dental degree	18.5 ± 12.2
Employment setting	
Sole proprietor	308 (43.7)
Shareholder owner in incorporated dental practice	135 (19.2)
Associate in incorporated dental practice	110 (15.6)
Other (such as an employee)	152 (21.6)
Personal net income (2006 dollars)	
≤$200,000	455 (68.8)
$200,001 – $400,000	162 (24.5)
> 400,001	44 (6.7)
Effort	
Hours worked chair side per week	31.0 ± 7.7
Weeks worked per year	45.9 ± 8.2
**Dental practice characteristics**	
Personnel	
Number of dentists other than respondent	2.3 ± 9.2
Dental hygienists	2.6 ± 7.5
Dental assistants	4.1 ± 11.3
Number of operatories available for restorative services	3.9 ± 10.2
Patients seen by respondent per week	52.0 ± 30.3
Pregnant patients seen by respondent per month	2.8 ± 4.4
Percentage of all patients with private dental insurance	61.7 ± 23.6
Respondent accepts capitation fees (yes)	155 (23.7)
Percentage of patients with Medicaid (government) dental insurance	11.1 ± 22.5
Percentage of Medicaid patients on capitation	30.1 ± 35.8
Capitation improves financial stability (1–5; 5 = strongly disagree)	3.9 ± 1.1
Age distribution of patients seen by respondent (percent)	
< 20 years	25.5 ± 15.7
20 - 44 years	41.5 ± 14.9
≥ 45 years	34.1 ± 16.7
Average patient wait in reception (1=<5 minutes; 2=5-15 minutes; 3=16-30 minutes; 4=>30 minutes)	1.4 ± .5
<5 minutes	420 (60.3)
5-15 minutes	263 (37.7)
16-30 minutes	13 (1.9)
>30 minutes	1 (.1)
Typical lead time to schedule new patient exam (1=1-2 days; 2=3-7 days; 3=1-2 weeks; 4=>2 weeks)	2.8 ± 1.0
1 or 2 days	67 (9.6)
3 days to a week	219 (31.4)
1 or 2 weeks	228 (32.7)
More than 2 weeks	184 (26.4)

^1^ For some variables, the number of respondents does not sum to 732 because of missing data.

### Counseling patients about maternal transmission of *Streptococcus mutans* and ECC

**Figure 1:**
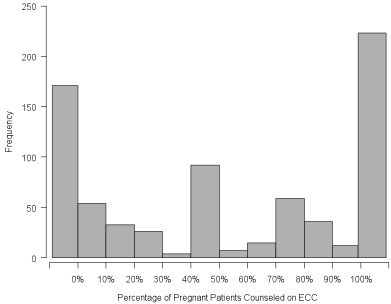
**Frequency of pregnant patients receiving counseling on ECC in Oregon State, USA in 2006.** Mean=53.5%; SD=41.4, *N*=732 dentists.

Nearly one-third (31%) of dentists reported they provided counseling on maternal transmission of *Streptococcus mutans* to all their pregnant patients; 46 percent reported they provided it to between 1 and 99 percent of pregnant patients and 23 percent reported they never discuss this with pregnant patients (Figure 1).

Table 2 displays characteristics of dentists who reported providing counseling to none, some, and all of their pregnant patients. Dentist personal and practice characteristics that were significant in the multinomial regression model are summarized, along with the four county-level characteristics under study.

**Table 2:** **Characteristics of dentists who provide ECC counseling to none, some and all pregnant patients (*N*=732)**

	**No.**^**1**^**(%) or Mean ± SD**				***P*value**
	**All dentists n=732**	**Counsels 0% n=171**	**Counsels 1-99% n=338**	**Counsels 100% n=223**	
**Personal/Practice characteristics**					
Experience providing dental services (years)	18.4 ± 12.2	21.6 ± 11.0	16.4 ± 12.1	19.0 ± 12.6	<.001
Medicaid patients (%)	11.1 ± 22.5	4.4 ± 14.4	13.1 ± 24.4	13.4 ± 24.0	<.001
Discuss ECC with colleagues (% yes)	331 (47.6)	36 (22.9)	162 (49.5)	133 (63.0)	<.001
ECC brochures directly to patients (% yes)	316 (43.2)	43 (25.2)	161 (47.6)	112 (50.2)	<.001
The link between caries in mothers and babies is too tenuous for me to warn my patients (1=strongly agree; 5=strongly disagree)	3.9 ± 1.0	3.3 ± 1.0	4.0 ± 1.0	4.3 ± 1.0	<.001
It is worth my time to counsel pregnant patients about how tooth decay can affect their baby (1=strongly agree; 5=strongly disagree)	1.8 ± 1.0	2.2 ± 1.0	1.7 ± 0.9	1.5 ± .9	<.001
**2007 Area Resource File Variables**					
Dentists/population rescaled	2.7 ± 1.0	2.7 ± 1.0	2.8 ± 1.0	2.7 ± 1.0	.51
Percent female dentists	19.7 ± 7.5	19.4 ± 7.8	20.1 ± 7.2	19.4 ± 7.8	.49
Percent females of childbearing age	20.1 ± 1.4	20.1 ± 1.4	20.1 ± 1.4	19.9 ± 1.5	.23
Percent in poverty	13.0 ± 2.9	12.9 ± 2.9	13.1 ± 3.0	12.9 ± 2.9	.73

^1^ For some variables, the number of respondents does not sum to 732 because of missing data.

### Impact of county-level characteristics on counseling on ECC transmission

Table 3 provides the results of the multinomial logistic regression in which we compared the three counseling groups, and report odds ratios for: 1) no counseling versus counseling 100 percent of pregnant patients and 2) some counseling (1-99% of patients counseled) versus counseling 100 percent of pregnant patients. The point estimates can be interpreted as the odds ratio of a dentist counseling less than 100 percent of pregnant patients after adjusting for the effects of dentist and practice characteristics and dentist beliefs.

**Table 3:** Multinomial logistic regression: impact of county-level characteristics of dentists on the percentage of pregnant patients receiving counseling on ECC transmission in Oregon State, USA

**Variable**	**Odds ratio (95% confidence interval)**	
	**0% v 100%**	**1-99% v 100%**
**Dentist and practice level characteristics and dentist attitudes and beliefs**		
Gender (0=female; 1=male)	2.3 (.8 - 6.2)	1.1 (.6 - 2.0)
Years providing dental services (10 years)	.9 (.7 - 1.3)	.8 (.6 - 1.0)**
Employment status (1= sole prop, partner or shareholder/owner; 0=anything else)	2.0 (.8 – 4.9)	1.1 (.6 - 2.0)
Number of hygienists	1.0 (.8 - 1.4)	1.2 (1.0 - 1.4)
Number of operatories	.8 (.7 - 1.1)	1.0 (.8 - 1.1)
Percentage of patients with Medicaid: (1 = 1-10%; 0 = 0%)	.6 (.3 - 1.4)	.8 (.4 - 1.4)
Percentage of patients with Medicaid: (1 = more than 10%; 0 = 0%)	.2 (.05 - .8)**	1.1 (.5 - 2.5)
Capitation payments improve financial stability of dental practices (1–5; 1=strongly agree; 5=strongly disagree)	1.1 (.8 - 1.5)	.9 (.7 - 1.1)
Percentage of patients younger than 15 years	1.0 (1.0 - 1.0)	1.0 (1.0 - 1.0)
Number of continuing dental education (CDE) courses (last 2 years)	1.0 (1.0 - 1.0)	1.0 (1.0 - 1.0)
Frequency of staff meetings (1=regularly; 2=occasionally; 3=never)	.6 (.3 - 1.1)	.7 (.4 - 1.2)
Average patient wait in reception room (1=<5 minutes; 2=5-15 minutes; 3=16-30 minutes; 4=>30 minutes)	1.2 (.6 - 2.3)	1.1 (.7 - 1.8)
Typical lead time to schedule new patient exam (1=1-2 days; 2=3-7 days; 3=1-2 weeks; 4=>2 weeks)	.9 (.6 - 1.3)	1.0 (.8 - 1.4)
CDE on Early Childhood Caries (ECC) educationist the last year (1=yes; 0=no)	.5 (.3 - 1.0)	.8 (.4 - 1.3)
ECC discussed with staff or colleagues (1=yes; 0=no)	.3 (.1 - .6)***	.8 (.4 - 1.3)
ECC brochures are given directly to patients (1=yes; 0=no)	.4 (.2 - .9)**	1.2 (.7 - 2.0)
Evidence for ECC transmission is tenuous (1–5; 1=strongly agree; 5=strongly disagree)	.5 (.3 - .7)***	.9 (.6 - 1.2)
It is worth my time to counsel pregnant patients on ECC (1–5: 1=strongly agree; 5=strongly disagree)	1.6 (1.1 - 2.3)**	1.5 (1.0 - 2.0)**
Control over your clinical decisions (1–10: 1=not important; 10=very important)	1.0 (.7 - 1.4)	.9 (.7 - 1.2)
Frequency of providing oral hygiene instruction to pregnant patients (1= often; 0=not often	.8 (.3 - 2.0)	.6 (.3 - 1.2)
Feelings about trying new procedures (1=enjoy experimenting with new procedures; 0=other)	.8 (.3 - 1.9)	.8 (.4 - 1.6)
**County level factors from the area resource file**		
Dentists/population 2007 rescaled	1.0 (.6 - 1.8)	1.0 (.7 - 1.6)
Percentage of female dentists 2007	1.0 (1.0 - 1.1)	1.0 (1.0 – 1.1)
Percentage females of childbearing age 2007	1.1 (.9 - 1.5)	1.2 (1.0 - 1.4)
Percentage in poverty 2007	1.1 (1.0 - 1.2)	1.0 (1.0 - 1.1)

** p <.05; *** p<.01.

The results show that, all other things equal, county-level factors (dentist/population ratio, percentage of female dentists, percentage of females of childbearing age, and percentage of the population living in poverty) had no independent effect on counseling to reduce ECC disease transmission.

## Discussion

The knowledge-building framework that guided these analyses posits that a greater number of dentists relative to the population of community results in increased counseling because of more frequent interaction between dentists and their social context. Similarly, we argued that a greater proportion of women in their child bearing years in the community would lead to more counseling because of increased demand for information. Neither hypothesis was supported.

Our previous work has shown that dentists' knowledge is systematically related to their estimates of the proportion of pregnant patients in their practice [20]. The results of the present study are striking because concerns about the increasing rates of ECC in the USA have already been discussed in the professional literature [6] and the evidence of maternal transmission risk and the effectiveness of prevention has been known for decades [21]. Yet, consistent with the diffusion of innovation literature in medical care, many dentists lag in changing their practices. Moreover, we have shown that dentists who do not believe the evidence about ECC transmission also do not provide education to their staff about how to address this problem. The social context of the dental office can affect knowledge transfer. In a study of the employees of a large staff-model dental care organization most dentists and dental hygienists responded correctly to knowledge questions about maternal oral health [22]. These practitioners may have been less isolated than individuals practicing in other settings because the organization made major investments in professional development and provider-patient communication.

Our previous report of the survey data examined in the present study documented that female dentists were more likely to counsel pregnant patients both about ECC disease transmission risk [7] and about periodontal health during pregnancy [11]. Our test for a similar association, using county-level provider data, found no significant association between the proportion of women dentists and the provision of counseling after adjusting for individual and practice level characteristics. This may be because, in the State of Oregon overall, less than one-in-five dentists are female and many counties outside of urban Portland have few women dentists. One hopes the counseling practices of women dentists will influence, through professional interaction, the behavior of all dentists. We have previously argued that there are opportunities to speed adoption by making continuing dental education on topics related to maternal oral health more available [14]. There is little evidence that the situation has changed markedly nationwide since these data were first collected and published.

Dentists who reported seeing a greater proportion of patients served by Medicaid government insurance for the poor were less likely to counsel their patients about ECC disease transmission (Table 3). In a previous paper about this dentist population, we showed that dentist output productivity varied with the proportion of Medicaid recipients in the practice, particularly for non-owners [23]. Thus, it may not be a surprise that counseling behavior is not influenced by community-level poverty even though low-income individuals are at a greater risk for maternal dental disease and ECC. The data suggest that the Oregon Medicaid program, indeed likely the programs in all States need to continue to raise their expectations of dentists’ behavior in this regard.

There are some limitations of this paper. First, this is a secondary analysis of a dataset collected for another objective, thus the questions available on the topic of ECC and disease transmission are limited. Second, the county may be too large and not represent the market area of the dental practice and thus the variables may have been measured at too gross a level to fully capture their influences on the dentists and their practices. Third, we did not analyze all potential county-level variables. While the number of OB/GYN specialists was available for 2007 and might reflect referrals to the dentists for care, this variable would not include the family practice physicians and nurse midwives delivering babies in many rural counties where there are no or few specialists. As such, the influence of factors such as the availability of pregnancy care providers may not be fully captured. Nevertheless, we conducted an analysis including this factor but the result was not significant (*Ps=*.98, 1.0*)*. Finally, the data were collected in 2006 and in only one state. Change may have occurred and Oregon State may not be typical of a large and diverse country as the USA. Nevertheless, research on this topic is scarce and the does provide insights into the functioning of the dental care delivery system in one state.

## Conclusions

In this study of Oregon, USA general dentists, we examined individual and practice level factors regarding counseling pregnant patients about the risks of maternal ECC disease transmission. We postulated, but were unable to show, any effect of county-level characteristics of the community on dentists’ report of this risk-prevention behavior.

## Competing interests

The authors declare that they have no competing interests.

## Authors’ contributions

PM was principal investigator of the research, conceptualized the paper, and wrote the final paper. CEH contributed to the conceptualization, helped define the variables, and contributed to the manuscript. LAM had primary responsibility for the statistical analyses and contributed to the manuscript. DLC wrote the conceptual framework for the paper and contributed to the overall manuscript. GG conducted the statistical analyses, incorporated the ARF variables, and prepared the tables and figure for the manuscript. DG was dual principal investigator for the research and contributed to the analysis and writing of the manuscript. All authors read and approved the final manuscript.

## Pre-publication history

The pre-publication history for this paper can be accessed here:

http://www.biomedcentral.com/1472-6831/13/23/prepub
